# Osmotic Demyelination Syndrome due to Rhabdomyolysis and Hyperosmolar Hyperglycemic Syndrome following Cardiogenic Shock

**DOI:** 10.1155/2021/8083731

**Published:** 2021-11-25

**Authors:** Kosuke Katano, Nozomi Fuse, Yoshitaka Asano, Kimihiro Osada, Akira Miyabe, Ryuma Ishihara, Atsushi Tosaka, Yuriko Satoh, Masako Maeda, Taisuke Mizumura, Akio Oshima, Toshitake Tamamura, Yoichi Sugimura

**Affiliations:** Kawakita General Hospital Cardiovascular Center, 1-7-3, Asagaya-kita, Suginami-ku, Tokyo, Japan

## Abstract

Osmotic demyelination syndrome (ODS) is a relatively rare disease that causes rapid demyelination, resulting in pontine and central nervous system damage with various symptoms, including impaired consciousness. It often occurs when hyponatremia is rapidly corrected. However, it can also occur when a normonatremic patient suddenly develops hypernatremia. A 51-year-old man developed cardiogenic shock with impaired consciousness, hyperCKemia, hypernatremia, and hyperglycemia. Osmotic demyelination syndrome secondary to rhabdomyolysis and hyperosmolar hyperglycemic syndrome was suspected. The patient's fluid volume decreased because of osmotic diuresis caused by hyperglycemia, and the blood sodium level increased rapidly. The latter resulted in ODS, which in turn resulted in a prolonged disturbance of consciousness, from which he has not yet recovered. ODS has been reported as a serious complication of rapid correction of hyponatremia, although it also occurs when normonatremia leads to hypernatremia. This disease is difficult to diagnose, as magnetic resonance imaging (MRI) of the brain is often unremarkable several weeks after its onset. This case of ODS occurred when normonatremia led to hypernatremia, as a result of rhabdomyolysis and hyperosmolar hyperglycemic syndrome. Diagnosis was made based on the MRI brain findings.

## 1. Introduction

Osmotic demyelination syndrome (ODS) is a relatively rare disease that causes rapid demyelination, resulting in pontine and central nervous system damage, with various symptoms including impaired consciousness. It often occurs when hyponatremia is rapidly corrected but can also occur when a patient with normonatremia suddenly develops hypernatremia. Although brain MRI findings are extremely important for diagnosis, they often do not reflect these changes in the acute phase, thus, making diagnosis difficult. We report the case of a normonatremic patient with ODS that occurred when he became hypernatremic due to rhabdomyolysis and hyperosmolar hyperglycemic syndrome, following cardiogenic shock. The diagnosis was made using the brain magnetic resonance imaging (MRI) findings.

### 1.1. Case Presentation

**Figure 1:**
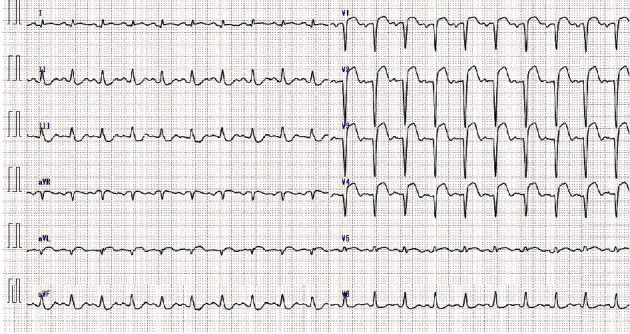
Electrocardiogram showing ST-segment elevation in V1-4.

**Figure 2:**
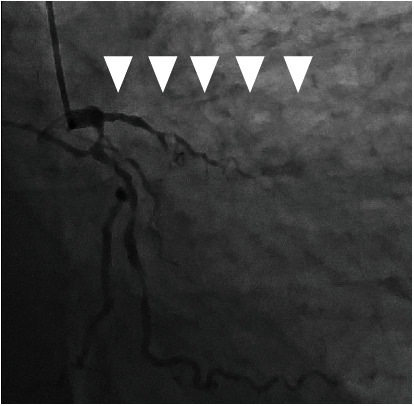
Left anterior descending artery occlusion (arrow) as identified by CAG. The left circumflex artery and high lateral branch of the coronary artery show diffuse stenosis. CAG: coronary angiography.

A 51-year-old man (height, 1.75 m; body weight, 83.1 kg; body mass index, 27.1 kg/m^2^) became aware of epigastric pain after dinner and reported to our hospital the next day with aggravated pain. The patient had a history of smoking (one pack per day). Moreover, he had a history of hypertension, diabetes mellitus, and hyperlipidemia; no family history of any other disease was noted. An electrocardiogram revealed ST-segment elevation in V1-4 (Figure 1); thus, we suspected myocardial infarction. Thereafter, we performed an emergency coronary angiography examination, which revealed a left anterior descending artery occlusion (Figure 2). Then, we attempted ad hoc percutaneous coronary intervention (PCI). Immediately after PCI was initiated, the patient developed acute heart failure, cardiogenic shock, and subsequent cardiopulmonary arrest. The cause of shock was acute left heart failure due to a sudden drop in cardiac output caused by myocardial infarction. Because of the occlusion of the proximal part of the left anterior descending branch, the perfusion area was extensive, and as a result, pulmonary edema progressed rapidly, resulting in cardiogenic shock.

He was immediately resuscitated with chest compressions, endotracheal intubation, percutaneous cardiopulmonary support (PCPS), and intra-aortic balloon pumping (IABP). PCI was successfully completed. He was weaned from PCPS and IABP on days 2 and 3 following the procedure, as his circulatory system was stable; he was extubated on day 6. Subsequently, impaired consciousness, hyperCKemia, hypernatremia, and hyperglycemia were observed.

#### 1.1.1. Impaired Consciousness

During intubation, midazolam, fentanyl, and rocuronium were administered continuously for sedation, analgesia, and muscle relaxation, respectively. Administration was discontinued after extubation; however, the patient's impaired consciousness persisted. On day 58, the patient was transferred to a convalescent hospital; at that time, his consciousness was in the range of Japan Coma Scale III-100–300.

**Figure 3:**
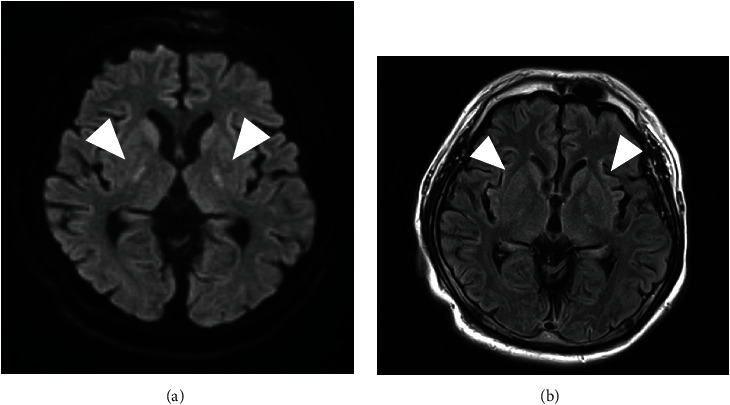
MRI on day 10 revealed only symmetrical hyperintensity in the putamen and posterior limb of the internal capsule (arrow). (a) DWI; (b) FLAIR. DWI: diffusion-weighted image; FLAIR: fluid-attenuated inversion recovery; MRI: magnetic resonance imaging.

**Figure 4:**
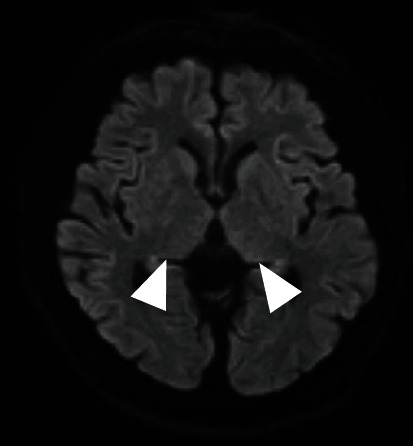
MRI on day 16 revealed no changes in the findings except loss of hyperintensity on DWI (arrow). DWI: diffusion-weighted image; MRI: magnetic resonance imaging.

**Figure 5:**
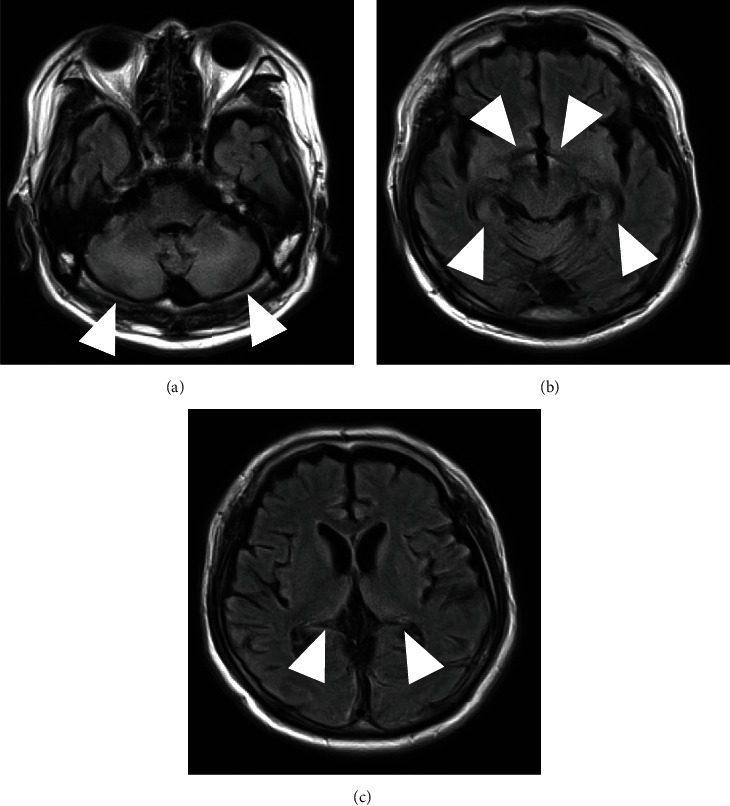
MRI on day 57 revealed symmetrical hyperintensity in the bilateral pons, cerebellar hemispheres, hippocampus, thalamus, and hypothalamus on FLAIR. (a) Cerebellar hemispheres (arrow). (b) Hippocampus and hypothalamus (arrow). (c) Thalamus (arrow). FLAIR: fluid-attenuated inversion recovery; MRI: magnetic resonance imaging.

An electroencephalogram was performed to investigate the cause of his diminished consciousness; no abnormal findings, such as epileptic waves, were observed. On day 10 following transfer, brain MRI findings revealed only symmetrical hyperintensity in the putamen and posterior limb of the internal capsule on axial diffusion-weighted imaging (DWI) and fluid-attenuated inversion recovery (FLAIR) (Figure 3). Hypoxic encephalopathy was ruled out because there were no findings in the thalamus or basal ganglia. Another MRI examination, which was performed on day 16, revealed no change in the findings except for hypointensity on DWI (Figure 4). On day 57 following transfer, the bilateral pons, cerebellar hemispheres, hippocampus, thalamus, and hypothalamus were symmetrically hyperintense on FLAIR findings (Figure 5). Other diseases that have persistent hyperintensity on FLAIR are cerebral infarction and posterior reversible encephalopathy syndrome (PRES). We ruled out cerebral infarction after magnetic resonance angiography findings confirmed that there was no lesion in the cerebral arteries; the findings were in areas that did not follow the vascular distribution. In addition, PRES was ruled out because the patient did not have severe hypertension, and the MRI findings did not improve even though the blood pressure remained stable after the onset of consciousness disorder. Thus, we diagnosed the presence of a consciousness disorder due to ODS.

#### 1.1.2. HyperCKemia

**Figure 6:**
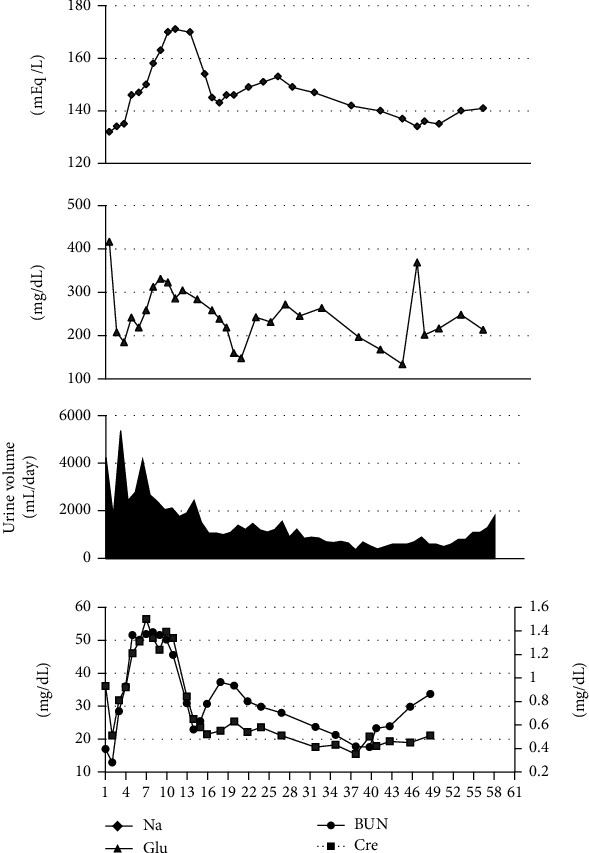
Clinical course of the patient concerning the levels of sodium, blood glucose, urine volume, BUN, and Cre. BUN: blood urea nitrogen; Cre: creatinine.

At presentation, his creatine phosphokinase (CPK) level was already elevated at 3,985 IU/L (Figure 6). Myocardial injury due to the anteroseptal infarction was severe and CPK peaked at 9,695 IU/L after 12 h. It decreased to the normal range (45–165 IU/L) on day 3, but increased again on day 4, reaching 22,830 IU/L on day 16. Hematuria was observed with the reelevation of CPK. The urinary myoglobin level (normal range: 0–10 ng/mL) was abnormally high at 26,000 ng/mL, indicating myoglobinuria. Therefore, we hypothesized that the increase in CPK after day 4 was attributed to rhabdomyolysis. The patient had no history of Parkinson's disease, muscle disease, or trauma. The rhabdomyolysis was considered drug-induced, most likely due to rocuronium.

### 1.2. Hypernatremia and Hyperglycemia

The level of sodium in the blood (normal range: 136–145 mEq/L) increased rapidly after day 4 and rose to 170.5 mEq/L on day 10. The blood glucose level was controlled with an insulin sliding scale until day 10, but the random blood glucose level remained at 300–400 mg/dL. Thus, insulin administration was continued. We diagnosed him with hyperosmolar hyperglycemic syndrome (HHS) because he had type 2 diabetes mellitus but had no acidosis or urinary ketone bodies. The urine volume was 3,000–5,000 mL/day, indicating polyuria. The urine osmolality (normal: 285 ± 5 mOsm/kg) was approximately 1,000 mOsm/kg, and the urine sodium level was approximately 70 mEq/L (normal range: 110–250 mEq/L), suggesting that osmotic diuresis associated with hyperglycemia was the cause. The patient's fluid volume decreased due to osmotic diuresis caused by hyperglycemia, and the blood sodium level increased rapidly, resulting in ODS, which in turn resulted in a prolonged disturbance of consciousness. Unfortunately, the patient's consciousness has not improved even after approximately 100 days.

## 2. Discussion

Rhabdomyolysis is a condition involving rapid breakdown and necrosis of skeletal muscle cells. Muscle cellular components are released into the bloodstream and extracellular space, causing acute renal injury and other sequelae [1]. Theoretically, any form of muscle damage and, by extension, any entity that causes muscle damage can initiate rhabdomyolysis. In adults, the available data show that the most common causes of rhabdomyolysis are drug or alcohol abuse, medicinal drug use, trauma, neuroleptic malignant syndrome, and immobility [2]. Although depolarizing muscle relaxants, such as succinylcholine, are frequently reported to cause rhabdomyolysis (drug-induced) [3], nondepolarizing muscle relaxants, such as rocuronium, may also be responsible.

It is known that rhabdomyolysis and HHS may be related. Some studies have shown a linear positive correlation between the serum CPK level, blood sodium concentrations, and plasma osmolality [4]. The pathogenesis of rhabdomyolysis associated with hyperglycemia remains unclear. However, it includes impaired glucose utilization due to insufficient insulin action, electrolyte abnormalities (i.e., hypokalemia and hypernatremia), extreme dehydration, impaired peripheral circulation, and impaired myocytic adenosine triphosphate production [5]. In this case, the osmotic diuresis caused by hyperglycemia decreased the amount of free water in the body, causing rapid progression of hypernatremia and subsequent ODS. This disease has been reported as a serious complication caused by the rapid correction of hyponatremia [6]; however, some studies have reported that it also occurs when normonatremia leads to hypernatremia [7], suggesting that osmotic differences may be involved in the pathogenesis. Although the pathogenesis remains unclear, it has been reported that the accumulation of microglia induces inflammatory cytokines and inducible nitric oxide synthase, promoting demyelination [8], and that the apoptosis of oligodendrocytes is involved [9]. Most cases of ODS involve central pontine myelinolysis based on the MRI findings in the center of the pons; however, in recent years, extrapontine myelinolysis, in which findings are noted in the basal ganglia, thalamus, and internal capsule, has also been considered as part of ODS. T2-weighted images and FLAIR findings show symmetrical high signals in those areas, and there is often no abnormality several weeks after onset [10].

Hypoxic encephalopathy is a disease, in which symmetrical high signals in the cortex, basal ganglia, and thalamus are observed on T2-weighted images. However, in this case, there were no such findings in the acute phase. Encephalopathy was ruled out since the patient was successfully intubated and ventilated immediately after respiratory arrest. Although a recent study showed that neurological prognosis was improved in approximately half of the patients [11], and there is evidence for the efficacy of immunosuppressive drugs [12], there is no established treatment for the disease to date and prevention is important. Hyponatremia has also been suggested as a poor prognostic factor, and the case caused by hypernatremia remains unclear [12]. In this case, hypernatremia occurred in a patient that was previously normonatremic, which is considered rare. Therefore, ODS should be considered early in patients with impaired consciousness who have abnormal blood sodium levels, and efforts should be made to prevent its occurrence.

## Data Availability

Raw data were generated at Kawakita General Hospital. Derived data supporting the findings of this study are available from the corresponding author on request.
